# A scoping review on tsetse fly blood meal sources and its assay methods since 1956 to 2022

**DOI:** 10.1186/s13071-023-06114-3

**Published:** 2024-02-02

**Authors:** Erick Kibichiy Serem, David Mwangi Mburu, Osman Abdikarim Abdullahi, Joel Ltilitan Bargul

**Affiliations:** 1 Department of Anatomy and Physiology, School of Health and Human Sciences, Pwani University, P.O. Box 195-80108, Kilifi, Kenya; 2 Pwani University Bioscience Research Centre (PUBReC), Pwani University, P.O. Box 195-80108, Kilifi, Kenya; 3 Department of Biological Sciences, School of Pure and Applied Sciences, Pwani University, P.O. Box 195-80108, Kilifi, Kenya; 4 Department of Public Health, School of Health and Human Sciences, Pwani University, P.O. Box 195-80108, Kilifi, Kenya; 5 Department of Biochemistry, Jomo Kenyatta University of Agriculture and Technology (JKUAT), P.O. Box 62000-00200, Nairobi, Kenya

**Keywords:** *Glossina* species, Blood meal sources, African trypanosomiasis, Vector-borne disease control

## Abstract

**Background:**

Tsetse flies (*Glossina* spp.) are the definitive biological vectors of African trypanosomes in humans and animals. Controlling this vector is the most promising method of preventing trypanosome transmission. This requires a comprehensive understanding of tsetse biology and host preference to inform targeted design and management strategies, such as the use of olfaction and visual cues in tsetse traps. No current review exists on host preference and blood meal analyses of tsetse flies.

**Methods:**

This review presents a meta-analysis of tsetse fly blood meal sources and the methodologies used to identify animal hosts from 1956 to August 2022. The Preferred Reporting Items for Systematic Reviews and Meta-Analyses extension for Scoping Reviews (PRIMA-ScR) was applied. This focused on tsetse-endemic countries, blood meal analysis methodologies and the blood meal hosts identified. The articles were retrieved and screened from databases using predetermined eligibility criteria.

**Results:**

Only 49/393 of the articles retrieved matched the inclusion criteria. *Glossina*'s main hosts in the wild included the bushbuck, buffalo, elephant, warthog, bushpig and hippopotamus. Pigs, livestock and humans were key hosts at the domestic interface. The least studied species included *Glossina fuscipleuris*, *G. fusca, G. medicorum, G. tabaniformis* and *G. austeni*. In the absence of preferred hosts, *Glossina* fed opportunistically on a variety of hosts. Precipitin, haemagglutination, disc diffusion, complement fixation, ELISA and PCR-based assays were used to evaluate blood meals. Cytochrome b (*Cyt b*) was the main target gene in PCR to identify the vertebrate hosts.

**Conclusions:**

Tsetse blood meal sources have likely expanded because of ecological changes that could have rendered preferred hosts unavailable. The major approaches for analysing tsetse fly blood meal hosts targeted *Cyt b* gene for species identification by Sanger sequencing. However, small-fragment DNAs, such as the mammalian 12S and 16S rRNA genes, along with second- and third-generation sequencing techniques, could increase sensitivity for host identification in multiple host feeders that Sanger sequencing may misidentify as “noise”. This review of tsetse fly blood meal sources and approaches to host identification could inform strategies for tsetse control.

**Graphical Abstract:**

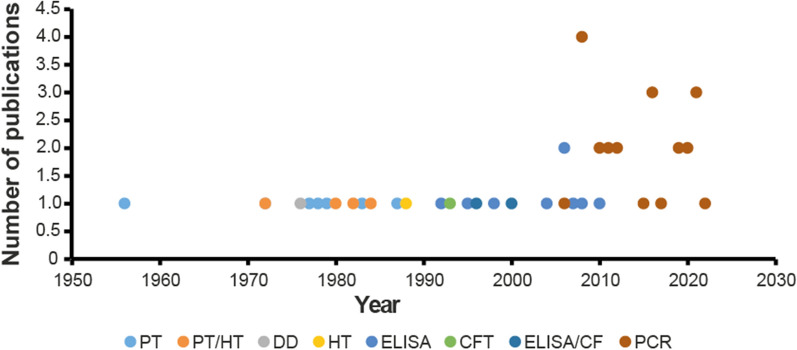

## Background

Tsetse flies (*Glossina* spp.) are obligate haematophagous biting insects that are endemic to 38 countries in sub-Saharan Africa. They are the definitive biological vectors of the African trypanosome parasites (genus *Trypanosoma*). Trypanosome parasites develop within the tsetse fly and transform into infective metacyclic trypomastigotes that cause trypanosomiasis in a wide range of mammalian hosts [1].

Tsetse flies digest blood meals in 1 ½ to 4 days, depending on the amount consumed [2], and they replenish their dwindling abdominal food reserves every 2 to 3 days [3]. Several approaches to analysing blood meals to decipher the hosts have been developed. These methods range from the traditional serological techniques such as precipitin test (PT) and enzyme-linked immunosorbent assay (ELISA) to an advanced molecular approach [4–6].

Several studies on tsetse fly blood meal hosts have improved our understanding of the preferred animal hosts and the vector’s potential role in the epidemiology of African trypanosomiasis [7–11]. However, the feeding habits of tsetse fly vectors may change over time depending on the availability of hosts in their habitats. For example, opportunistic feeding behaviour in *Glossina palpalis* has been shown to shift from rhinoceroses to suids over time, probably because of ecological changes that made the preferred blood meal hosts unavailable [12]. It has been proposed that whenever an environmental change is detected, the feeding sources of tsetse flies may need to be assessed [13]. To surmount this, some of the limitations need to be overcome. For instance, the time taken from sample collection to publication of results on analysis of blood meals cited in the literature ranged from 1 to 16 years [12, 14]. Thus, by the time some studies are published, particularly those documented over a decade later, ecological changes may have occurred and the preferred blood meal hosts might have migrated to alternative locations.

To our knowledge, there is no current review of tsetse fly blood meal analysis and the methodologies used to determine the vector’s propensity for blood meals from potential animal hosts. Thus, we set out to (i) review the published literature on tsetse blood meal sources and methods used for analysis from 1956 to August 2022, (ii) identify tsetse fly species whose feeding preference(s) on hosts are less understood and (iii) identify the strengths and drawbacks of methods used for blood meal analysis.

## Methods

### Protocol and registration

The scoping review procedure for this study was submitted to the Open Science Framework (OSF) (https://osf.io/yczqp/). It was created using the Preferred Reporting Items for Systematic Reviews and Meta-Analyses extension for Scoping Reviews (PRIMA-ScR) criteria [15].

### Eligibility criteria

The set criteria for eligibility of the peer reviewed journal articles for this review included original research publications on tsetse blood meal analysis, articles written in English and articles published up to August 2022. We excluded experimental studies and those in which blood meal hosts were not determined.

### Article search strategy from information sources

The article searches were performed using PubMed, Scopus and International System for Agricultural Science and Technology (AGRIS) electronic databases. The key words used in the search for peer reviewed journal information included “blood”, “meal” and “tsetse”.

### Selection of sources of the evidence

**Fig. 1:**
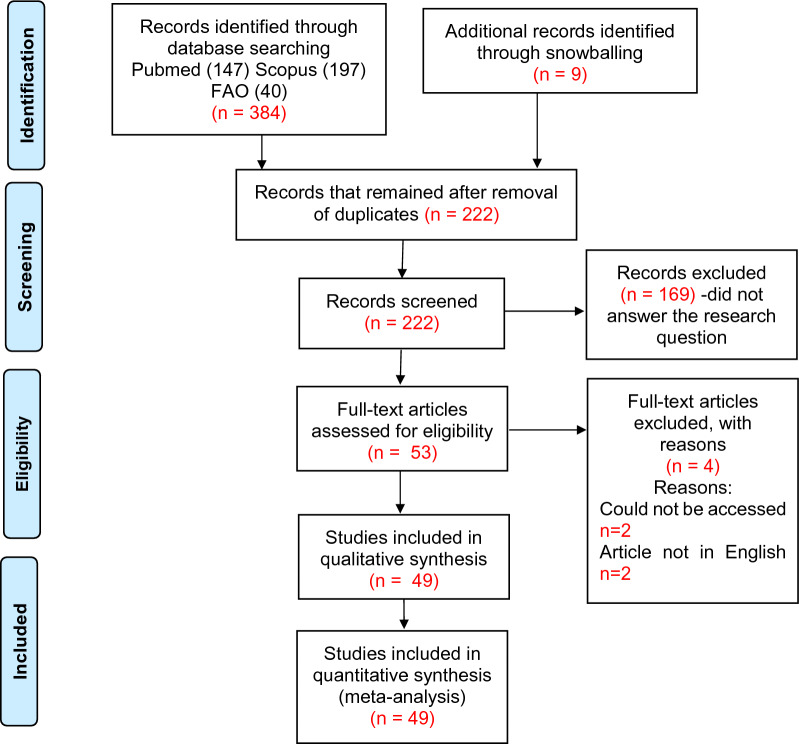
Flow chart showing the selection of articles for inclusion in the scoping review. A total of 393 articles were retrieved from PubMed, AGRIS FAO and Scopus electronic databases. After removal of duplicates, 222 articles were screened for relevance and ultimately 53 articles were included. Two articles were in French (abstracts were in English) and two could not be accessed because of subscription access. Finally 49 articles were included in the study.

The PubMed, Scopus and FAO-AGRIS databases were queried individually and fed into Rayyan artificial intelligence software (https://www.rayyan.ai/). The search results were evaluated and duplicates deleted. Titles and abstracts of the peer-reviewed articles were used to determine their eligibility. Two reviewers independently screened the selected articles for inclusion. All studies that did not answer the set research question(s) were disregarded. The selected full-text journal articles in Portable Document Format (PDF) were uploaded to Rayyan software for data extraction. A total of 53 articles were included for the full text screening and data charting (Fig. 1).

### Data extraction

Data extraction was performed using a data-charting sheet developed in this review (Additional file 1). The authors, title, year of publication, country, study area, period of sample collection, tsetse fly species studied, number of samples analysed, type of samples collected, diagnostic method used and proportion of samples positively identified were obtained from each publication. The gene and sequencing methods were extracted from articles on molecular approaches to blood meal host identification.

### Data analysis

Descriptive data analysis was conducted using R software version 4.3.2 and the results presented as frequencies and percentages. Where appropriate, the data were presented using graphs or tables.

## Results

### Publication statistics

The initial reference title searches yielded 384 relevant publications. These included 147, 197 and 40 publications from PubMed, Scopus and FAO-AGRIS, respectively. After removal of duplicates, a full text search was done on 213 selected references. Snowballing from the selected references yielded nine more publications, resulting in 222 articles (Fig. 1). The final synthesis included 49 publications (Table 1).

**Table 1:** Distribution of blood meal studies per nation in Africa

Species	Country	Blood meal method	Gene amplified	Sequencing technique	Period of data collection	Year of publication	References
*Glossina palpalis palpalis* *G. pallicera pallicera* *G. caliginea,* *G. nigrofusca*	Cameroon	PCR	*Cyt b*	Heteroduplex hybridization Sanger	2008	2010	[13]
*G. palpalis palpalis*				Heteroduplex hybridization	2006–2007	2011	[54]
*G. palpalis palpalis*					2012	2016	[55]
*G. palpalis palpalis,* *G. pallicera pallicera* *G. caliginea,* *G. nigrofusca*					2002–2004	2008	[29]
*G. palpalis palpalis*		ELISA			1999–2000	2004	[56]
*G. tabaniformis* *G. fuscipes fuscipes*	Central African Republic	PCR	*Cyt b*	Illumina	2012	2015	[49]
*G. fuscipes fuscipes* *G. palpalis palpalis*		CFT			1990	1993	[57]
*G. palpalis palpalis*	Congo	PCR	*Cyt b*	Heteroduplex hybridization	2009	2012	[58]
*G. fuscipes quanzensis*					2005	2006	[14]
*G. fuscipes quanzensis*					2005	2009	[59]
*G. longipalpis* *G. medicorum* *G. palpalis palpalis* *G. palpalis gambiensis*	Côte d'Ivoire	ELISA CFT			1988	2000	[10]
*G. pallidipes*	Ethiopia	PCR	*Cyt b*	Sanger	2016	2019	[16]
*G. morsitans submorsitans*		ELISA			2000–2001	2007	[60]
*G. palpalis palpalis* *G. fuscipes fuscipes* *G. caliginea*	Gabon	ELISA			2007	2010	[61]
*G. palpalis palpalis* *G. fuscipes fuscipes*		PCR	*Cyt b*, COI, 16S rRNA	Sanger	2012–2014	2017	[62]
*G. morsitans submorsitans*	Gambia	PT			1977	1979	[63]
*G. palpalis gambiensis,* *G. morsitans submorsitans*		ELISA CFT			1990	1996	[19]
*G. pallidipes,*	Kenya	PCR	*Cyt b*	Sanger	2012	2021	[27]
*G. pallidipes,* *G. swynnertoni*		PCR-HRMA	*Cyt b*, COI	Sanger	2016	2021	[28]
*G. pallidipes*		ELISA			2004–2005	2008	[18]
*G. pallidipes* *G. longipennis*					1992, 1990	1995, 1995	[64, 65]
*G. pallidipes* *G. fuscipes fuscipes*					NI	1997	[33]
*G. pallidipes*		HIT CFT			NI	1998	[66]
*G. pallidipes*		HIT			2005–2007, 1978–1981 1968–1970 1970–1971	1987, 1983, 1972, 1972	[67–70]
*G. palpalis gambiensis* *G. tachnoides*	Mali	PCR	*Cyt b*	Sanger	2008–2009 2008–2009	2010, 2013	[38, 39]
*G. morsitans morsitans* *G. pallidipes*	Mozambique	PCR	*Cyt b*	Sanger	2009–2014	2020	[71]
*G. palpalis* *G. tachinoides*	Nigeria	PCR	*Cyt b*	Sanger	2014	2016	[72]
		PCR	*Cyt b*	Sanger	2016–2017	2019	[40]
*G. morsitans* *G. swynnertoni*	Tanzania	PCR,	12S rRNA	Illumina	2012–2013	2022	[44]
*G. pallidipes* *G. m. morsitans* *G. brevipalpis*		PT HIT			1976–1978	1984	[8]
*G. swynnertoni* *G. pallidipes*		PCR	*Cyt b*	Sanger	2006	2016	[73]
*G. pallidipes* *G. fuscipes fuscipes* *G. brevipalpis*	Uganda	PT HIT			1969–1970	1980	[74]
*Glossinia fuscipes fuscipes*		ELISA			2000	2006	[34]
*G. fuscipes fuscipes*		PCR	*COI* *Cyt b*	Sanger	NI	2021	[75]
*G. pallidipes*	Zambia	PCR	*Cyt b*	Sanger	2006, NI	2008, 2008	[17, 76]
*G. morsitans morsitans*		PH, HIT			1974–1975	1982, 1977	[77, 78]
*G. pallidipes* *G. morsitans morsitans*	Zimbabwe	PT			1971	1978	[79]
*G. pallidipes* *G. morsitans morsitans*		Gel-diffusion			1974	1976	[80]
*G. morsitans centralis*	Zambia, Zimbabwe	PCR	12S rRNA	Illumina	2014, 2017	2020	[50]
*G. swynnertoni* *G. pallidipes*	Kenya, Tanzania, Uganda	PCR	*Cyt b*	Sanger	2008–2009	2011	[11]
*G. swynnertoni* *G. morsitans* *G. pallidipes* *G. austeni* *G. palpalis fuscipes* *G. brevipalpis* *G. longipennis*	Sudan, Uganda, Kenya, Tanzania South Africa	PT			1950–1955	1956	[81]
*G. fusca* *G. fuscipleuris* *G. brevipalpis* *G. longipennis* *G. palpalis* *G. fuscipes* *G. tachinoides* *G. morsitans* *G. longipalpis* *G. pallidipes* *G. austeni*	Central African Republic, Cote D’Ivore Kenya Uganda Somalia Zambia Bukina Faso Republic of Congo Gambia Liberia Senegal Zaire Mali Sudan Ethiopia	ELISA			1982–1993	1998	[12]

Summary of the blood meal analysis studies by country, tsetse fly species and method(s) used. A total of 12/49 (24.5%) articles were published between 1956 and 1990. Publications increased from 1990 to 2022. Most publications after 1990 reported ELISA or molecular diagnostic techniques. Kenya, Uganda, Zambia, Cameroon and Tanzania had 21.2% (14/66), 9.1% (6/66), 9.1% (6/66), 7.6% (5/66) and 7.6% (5/66) studies respectively

### Methods of identifying vertebrate hosts that tsetse feed on

**Fig. 2:**
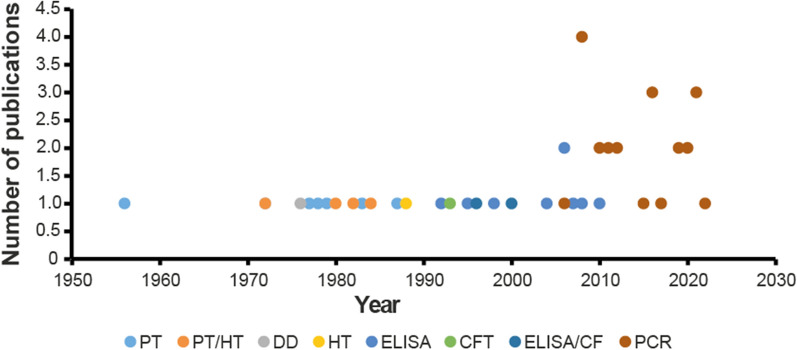
Scatter plot of studies per blood meal method (*n* = 49). Polymerase chain reaction (PCR), enzyme-linked immunosorbent assay (ELISA), haemagglutination inhibition test (HIT), precipitin Test (PT), gel diffusion (GD) and complement fixation test (CFT) were used. Of the 24/49 (49%) studies which utilized PCR, 1/24 (4.2%) used high-resolution melting (HRM) analysis and 1/24 (4.2%) used multiplex PCR

This review found that molecular detection of the host DNA was used in 77.4% (24/31) of the studies published after the year 2000. ELISA, precipitin test (PT), haemagglutination inhibition test (HIT), disc diffusion or a combination of PT and HIT or ELISA and complement fixation test (CFT) were utilized in studies conducted before 2005. The methods for analysing blood meals are summarized in Fig. 2.

**Fig. 3:**
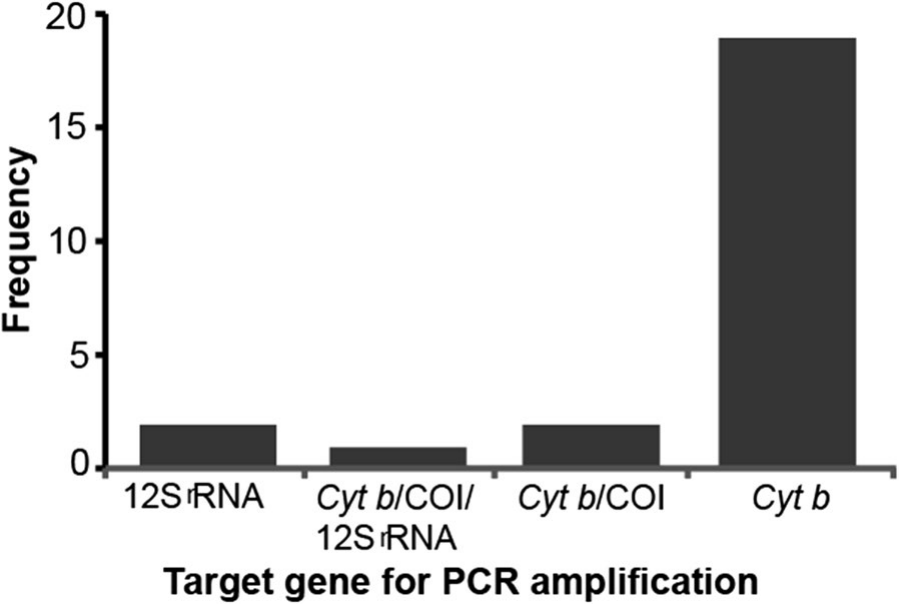
Distribution of gene-specific PCR studies for mammalian host blood meals (*n* = 24). Most of the studies amplified only *Cyt b* gene 19/24 (79.2%), 2/24 (8.3%) amplified both *Cyt b* and *COI*, 2/24 (8.3%) amplified 12S rRNA gene and 1/24 (4.2%) amplified a combination of the three genes

Some studies utilized PCR for amplification of the target gene, amplicon sequencing and comparison of the query sequences with information available in the GenBank of NCBI (BLAST) to identify hosts for tsetse blood meals. The mitochondrial cytochrome *b* (*Cyt b*) gene was targeted in 21/24 (87.5%) of the studies that employed PCR-based molecular assays. This gene is associated with limited intra-species variability and high inter-species variation. Figure 3 shows the distribution of different genes in the identification of tsetse fly blood meal sources. Sanger sequencing (*n* = 12/24; 50%) was the most commonly used method followed by heteroduplex hybridization (*n* = 8/24; 33.3%). Illumina second-generation sequencing accounted for 12.5% (*n* = 3/24). Only Cameroon (*n* = 4/7; 57.1%) and the Democratic Republic of Congo (*n* = 3/7; 41.3%) reported results using heteroduplex hybridization.

### Tsetse flies species and their blood meal sources

Blood meal hosts were identified in 20 of the 33 tsetse fly species and subspecies present in Africa (Table 2). *Glossina pallidipes* was the most studied species accounting for 21/87 (24.1%) of the published articles, followed by *G. palpalis palpalis* at 13.8% (13/87). *Glossina fusca*, *G. medicorum* and *G. austeni* were the least studied at 1/87 (1.1%), 2/87 (2.3%) and 2/87 (2.3%) respectively. Thus, determination of tsetse fly host feeding preferences through blood meal analysis of 13/33 (39.4%) *Glossina* species and sub-species may not have been fully evaluated.

**Table 2:** Major and minor blood meal hosts for *Glossina* spp.

Hosts preferred for blood meal by tsetse					
Tsetse fly species	No. of publications	Percentage %	Major hosts	Minor hosts	Reference(s)
Group I: *Glossina* feeding mainly on Suidae (*G. austeni and G. fuscipleuris*)					
* G. austeni*	2	2.3	Bushpig	Cattle	[12, 24]
* G. fuscipleuris*	1	1.1	Bushpig	Cattle	[12]
Group II: *Glossina* feeding equally on Suidae and Bovidae (*Glossina morsitans morsitans, G. m. submorsitans* and *G. m. centralis*)					
* G. m. morsitans*	11	12.6	Kudu, buffalo, bushbuck, bushpig, warthog, elephant	Waterbuck, hippopotamus, rhino, reptiles, baboon	[19, 60, 77, 80]
* G. m. centralis*	1	1.1	Human, cattle, dog, bushpig, African buffalo	Greater kudu, rat, bat, waterbuck	[50]
Group III: *Glossina* feeding mainly on Bovidae (*Glossina pallidipes, G. longipalpis* and *G. fusca*)					
* G. pallidipes*	21	24.1	Bushbuck, buffalo, greater kudu, elephant, human warthog, bushpig, hippopotamus	Chicken, mouse, goat, hyena, giraffe, antelope	[18, 27, 28, 71, 73]
* G. longipalpis*	1	1.1	Bushbuck, Duiker	Monitor lizard, hippopotamus and rodents, bushpig, cattle, dog, chicken	[10]
* G. fusca*	1	1.0	Bushbuck	Cattle, domestic pig, warthog	[12]
Group IV: Species feeding mainly on mammals other than the domestic pigs or Bovidae (Glossina *longipennis* and *G. brevipalpis)*					
* G. swynnertoni*	4	4.6	Buffalo, giraffe, warthog, elephant, human, hippopotamus	Hyena, ostrich, eland, baboon, Kori bustard, mice, waterbuck, pig, lion, duiker, gazelle	[28, 73]
* G. brevipalpis*	4	4.6	Hippopotamus, bushbuck, elephant,	Warthog, bushpig, waterbuck, cattle, buffalo	[12, 74]
* G. longipennis*	3	3.4	Bushpig, warthog, hippopotamus, buffalo, elephant, warthog	Rhino, dik-dik, ostrich	[12, 64]
Group 5: Species that fed on human and other available hosts (*Glossina palpalis*, *G. fuscipes* and *G. tachinoides*)					Reference
* G. p. palpalis*	12	13.8	Pigs, human, cattle, Nile monitor lizard	Sheep, dog, duiker monkey, sitatunga, golden cat, warthog, bushbuck, hippopotamus, wild birds, rodents	[13, 40, 54, 55, 62]
* G. f. fuscipes*	10	11.5	Buffalo, human, sitatunga, Nile monitor lizard, pig	Buffalo, sitatunga, duiker, bushpig, goat, African elephant, dog, African mud turtle	[49, 57, 61, 74, 75]
* G. tachinoides*	3	3.4	Human, cattle,	Spotted hyena, giraffe, crocodile, goat	[38–40]
* G. p. gambiensis*	4	4.6	Cattle, domestic pig, warthog	human, bushbuck, reptiles	[10, 19, 38, 39]
* G. medicorum*	1	1.1	Bushbuck	Cattle, human, crocodile, monitor lizard, warthog, bushpig	[10]
* G. f. quanzensis*	2	2.3	Human, pigs		[14, 59]
* G. pallicera*	2	2.3	Human, pig,	Goat, sheep, python	[13, 29]
* G. tabaniformis*	1	1 .1	Buffalo, human, sitatunga	Bushpig, duiker	[49]
* G. caliginea*	2	2.3	Buffalo Human, snake	Goat	[61, 62]
* G. nigrofusca*	2	2.3	Human	Sitatunga	[13]

Publications per tsetse fly species and their major and minor blood meal hosts. A host was designated as ‘major’ if ≥ 10% of the tsetse analysed fed on it and ‘minor’ if < 10%. *Glossina pallidipes* was the most studied species at 24.1%. *Glossina fusca, G. medicorum, G. longipalpis, G. tabaniformis, G. pallicera* and *G. austeni* were the least studied tsetse flies

The blood feeding habits of tsetse flies were influenced by their habitat. This study clustered tsetse flies by collection site. The first group involved ‘human-wildlife’ interface such as the protected national parks and game reserves, while the second group was ‘human-wildlife-domestic animals’ interface such as the edges of protected parks. The third group involved human-domestic-animals interface in human residential areas. Tsetse flies’ blood meal hosts were extracted and summarized by their preferred hosts as shown in Table 2.

## Discussion

This study focused on blood meal sources and the methods of blood meal analysis in tsetse flies from 1956 to August 2022. The blood meal sources were mainly dependent on the ecology and the species of tsetse fly vectors. Suids (warthogs, domestic and bush pigs) and bovids (bushbuck and buffalo) were the preferred hosts for most tsetse fly species. Precipitin and haemagglutination tests were the commonly used methods of blood meal analysis from 1956 to 1988. ELISA was predominantly utilized from 1992 to 2008. Most studies after the year 2001 utilized PCR. The traditional serological tests such as PT were less sensitive and could not identify all blood meal hosts of tsetse flies. However, molecular techniques were more sensitive and led to identification of more blood meal hosts.

Kenya recorded the highest number of published research studies focusing on tsetse fly blood meal analysis. This may be due to the existing vibrant research institutions such as International Livestock Research Institute (ILRI), International Centre of Insect Physiology and Ecology (*icipe*) and Kenya Agricultural and Livestock Research Organization (KALRO)—all of which collaborate in research with Kenyan institutions of higher learning. Additionally, Kenya has active government-led programmes on tsetse fly and trypanosomiasis eradication led by the Kenya Tsetse and Trypanosomiasis Eradication Council (KENTTEC) in conjunction with the aforementioned research institutes for adaptive and operational research.

Most of the studies focused on *Glossina pallidipes (24.1%), G. palpalis (13.8%)*, *G. morsitans (13.7%)* and *G. f. fuscipes* (11.5%) [16–19], which are considered as the main vectors of AAT and HAT in Eastern and Southern Africa and are the predominant species of tsetse flies [20–22]. *Glossina fuscipes* accounts for at least 90% of the HAT transmissions [23]. Regionally unlike the other species of tsetse flies, *G. austeni*, a vector of AAT, is present only on the east coast countries of the African continent including Somalia, Kenya, Tanzania, Mozambique and north-eastern parts of South Africa and Zimbabwe. From the published studies, only two studies were conducted on blood meal analysis for *G. austeni* from the tsetse fly samples that were collected between 1982 and 1993 [12] and one from sample data collected between 1950–1955 [24]. Since the Morsitans group have been identified as the major vectors of AAT, there is a need to carry out more blood meal analyses to check the possibility of varying hosts that can be fed on by Morsitans tsetse flies such as *G. austeni* and the resultant transmission of trypanosomiasis*.*

There seemed to be an expansion of tsetse flies feeding patterns likely guided by semiochemical cue attraction emanating from the bodies of preferred blood meal hosts from which tsetse flies fed on. The presence of these hosts may, however, be influenced by the ecosystem services and functioning. The grouping of tsetse flies into the five broad groups was based on the blood meal analyses as reviewed by Weitz [25]. Host expansion over time was noted in all the tsetse groups but notable changes in group I, III and V were observed. Among group I, *Glossina* that feed mainly on Suidae, *G. austeni*'s preference for suids seems to have expanded as recent studies identified the bushbuck as the preferred host [26]. This change could have been influenced by the availability of the vertebrate hosts. For group III, *G. pallidipes*, which mainly feed on Bovidae, other main hosts included bush pig, elephant and warthog [9]. Recent studies have identified other preferred hosts of *G. pallidipes* including hippopotamus, human, giraffe, warthog and buffalo [18, 26–28], suggesting that different tsetse fly species may not discriminate the hosts so long as they get a blood meal.

Among group V, tsetse flies that feed on human and commonly occurring hosts including bovids, suids, reptiles, canines, elephants and crocodiles, the feeding patterns in this group varied from place to place. For example, in Cameroon, *G. palpalis* fed more on pigs than on human in Bipindi region yet in Campo region they fed more on humans than on pigs, with sheep and antelopes being minor hosts [29]. Moreover, other studies reported new hosts in the same area including the duiker, monkey and sitatunga [13].

In light of advanced molecular techniques, the tsetse blood meal analysis categories might need to be revised, particularly in terms of genetics, to ensure appropriate classification. Some studies on molecular phylogenetics of tsetse flies, for instance for Palpalis group, reported that the morphology of the female genital plates was used in their grouping. However, some individuals of the fly species were later found to vary outside the ranges that were specified in the standard identification/taxonomic keys, making definitive morphological grouping impossible [30]. The minimum period between collection of tsetse fly samples and blood meal analysis ranged from 1 to 16 years. Tsetse fly host searching behaviour for the exclusive blood meal feeding may be altered as a result of their differential host dependence in response to ecological changes [12]. Thus, it is important to characterize the feeding behavioural host dependence as defined by the presence of different hosts’ blood meals in a single fly in order to detect any potential changes in the epidemiology of AAT. Some studies have confirmed the change in the feeding preference of tsetse flies on hosts [13, 29].

### Serological Methods utilized in blood meal analysis

Serological techniques were the earliest methods to be used to unravel tsetse flies’ source of blood for their meals and survival. The serological techniques utilized for blood meal analysis in tsetse flies included precipitin test (PT), haemagglutination inhibition test (HIT), complement fixation test (CFT) and enzyme-linked immunosorbent assays (ELISA). The limitations of precipitin test method included inability to detect highly digested blood meal, and the small amounts of blood consumed by the flies led to a small amount of antigens available for testing [31]. HIT test was more sensitive than PT and thus was better at the identification of individual hosts [8]. The introduction of CFT in tsetse blood meal analysis improved the sensitivity and reliability of blood meal analysis but did not permit absorbance of antisera with cross-reacting antigens [7] and thus it may not be used to differentiate the closely related species. The introduction of ELISA further improved blood meal analysis because of its sensitivity and reliability. The advantages of the ELISA test in blood meal source analysis have been enumerated by Sakamoto and others [32], including the ability to identify mixed blood meals from different species. In addition, it has high specificity and sensitivity as well as high efficiency; it is a simple and eco-friendly procedure given that radioactive substances and large amounts of organic solvents are not required and it uses cheap reagents. However, some of the limitations identified included inability to detect all blood meal sources and decreased sensitivity of the test in highly digested blood meal or small quantities of it [33]. The requirement of specific antisera and cross-reactivity in related species [34] coupled with the need to have the antisera against all possible sources of blood meal in a given target area where tsetse flies are collected are also limiting factors [11, 35].

### PCR-based molecular assays

The development of highly sensitive DNA barcoding techniques has helped molecular biologists to understand the parasite *Trypanosoma*, the tsetse fly species and their hosts [36]. The barcoding process uses short standardized genomic DNA fragments such as COI and *Cyt b* genes to distinguish among species [37]. The amplification of the mitochondrial proteins *COI* and Cyt *b* has proven useful in identifying blood meal sources in tsetse flies [11, 13, 38–40].

*Cyt b* gene is smaller in size than *COI* gene at 400 bp and 650 bp, respectively. Both *Cyt b* and *COI* genes are variable between different organisms but invariable within the same species. Comparatively, *Cyt b* and *COI* are similar in identifying intra-species variation but *Cyt b* is better in the analysis of smaller mammalian fragment samples such as trace or degraded samples [41]. The latter is useful in field-collected samples of hungry tsetse flies with minimal residual blood in the gut. Crushing the insect samples in liquid nitrogen improves the DNA yields [42]. However, other studies have reported that liquid nitrogen may damage to genomic DNA [43]. The COI gene has been less reliable for use in blood meal analysis [27]; hence, most studies use *Cyt b* gene.

Recently, other genes have been identified and used in the identification of the blood meal hosts. For instance, 12S rRNA gene has been shown to be more sensitive in identifying hosts from highly degraded blood meals [29, 44]. The 150-bp region of the 16S rRNA gene has also been used to identify hosts from highly degraded blood meals where *Cyt b* gene could not be amplified [45].

With the onset of the utilization of molecular approaches, different sequencing techniques have been used for elucidating the blood meal sources in tsetse flies’ gut including hybridization, Sanger and Illumina [11, 14, 44]. Over time, sequencing techniques have improved tremendously. The first method was heteroduplex hybridization, which uses an ordered array of oligonucleotides to identity a target sequence [46]. This method was successfully used, albeit underlying limitations including the exclusion of repetitive sequences [47]. Hybridization was later replaced by the first-generation Sanger sequencing technique invented by Frederick Sanger in 1977 [48]. Second-generation sequencing techniques, Illumina and Roche 254, have been used to sequence PCR-based amplicons from blood meals in tsetse flies fed from an array of hosts [44, 49, 50].

Tsetse flies prefer to go back to the original vertebrate host species for consecutive blood meals, although during starvation it may feed on any available host [51]. However, second-generation sequencing suggested that most tsetse flies had blood meals from multiple hosts. For example, Gaithuma and colleagues in two tsetse endemic foci, Kafue in Zambia and Hurungwe in Zimbabwe, confirmed the multiple feeding behaviour of these vectors, including feeding upon four different hosts [44, 49]. This raises the question: do tsetse fly species have preferred host(s)? Additionally, since most of the sequencing for determination of hosts has been based on the Sanger sequencing, does this mean it only detects the most abundant blood meal? Furthermore, it is also unclear whether the “dirty” sequences, which are not processed for blast analysis, represent blood from multiple hosts.

Currently, there are second-generation sequencing techniques including 454 pyrosequencing, Illumina and Ion Torrent. Oxford Nanopore and Single Molecule Real Time (SMRT) are considered third-generation sequencing technologies. All these techniques have enriched the accuracy of throughput depth of our scientific understanding at the molecular level as reviewed by different authors [47, 52]. The second- and third-generation sequencing techniques have their benefits and limitations that usually allow for technology advancement. For instance, SMRT can process sequences faster rate with longer reads than the second-generation sequencers, although they can have a high error rate of up to 20% as reviewed by Bleidorn [52]. However, there is a need to further explore the concept of blood meal analysis using the second- and third-generation sequencing techniques, albeit their availability in the African context.

With real-time PCR and PCR coupled with high-resolution melting analysis (PCR-HRM), it is now possible to screen many samples with minimal blood meals in them and reduce the cost of sequencing all the positive samples when appropriate controls are used. PCR-HRM can accurately identify blood meal sources from tsetse flies, including those which fed on multiple hosts [28], and could help overcome the challenge faced with Sanger sequencing, which may not give a clear chromatogram especially in cases of mixed blood meals.

Although speculative, most of the Sanger sequences that are not ‘clean’ enough for BLAST analysis may be feeds from multiple hosts. We hypothesise that the use of PCR-linked HRM and the use of smaller fragments including 12S and 16S rRNA genes could considerably improve blood meal analysis. This is because of PCR-linked HRM's sensitivity in identifying multiple hosts and reducing the cost of sequencing, especially in the African context of limited resources.

### Limitation of the scoping reviews

Scoping reviews are important in addressing broad research questions and thus valuable in guiding whether a systematic review will be required. However, due to the breadth of the issue they cover, scoping review results are not conclusive and from the onset may swing towards an area of focus because of limitations in coverage and an absence of quality control unlike systematic studies [53]. This scoping review should therefore be interpreted in light of the above limitations.

## Conclusions

The current review mapped out studies on the blood meal analysis in African countries published in English. It found that about half of the African countries endemic for tsetse flies and trypanosomiasis have carried out blood meal analysis. In addition, host-seeking preferences of about two-thirds of the tsetse fly species have been studied. The advent of technological advancement including PCR methods for library preparation and high-throughput sequencing technology in diagnostics has seen old fashioned techniques such as precipitin tests replaced by deterministic molecular diagnostic methods. We recommend that future studies on host preferences of tsetse fly species should deploy small fragment DNA, such as the mammalian 12S and 16S rRNA genes amplified using PCR-HRMA, qPCR or dPCR to enhance efficiency in library preparation and to reduce sequencing costs. Second- or third-generation sequencing approaches are more likely to give accurate results, especially when the vectors have fed on multiple hosts, which may be misidentified by Sanger sequencing as background noise.

## Data Availability

All data generated or analysed during this study are included in this published article [and its supplementary information files].

## Abbreviations

AAT: Animal African trypanosomiasis
AGRIS: International System for Agricultural Science and Technology
BLAST: Basic Local Alignment Search Tool
CFT: Complement fixation test
COI: Cytochrome oxidase I
Cyt b: Cytochrome b
dPCR: Digital polymerase chain reaction
ELISA: Enzyme-linked immunosorbent assay
FAO: Food and Agricultural Organization
HAT: Human African trypanosomiasis
HIT: Haemagglutination inhibition test
ICIPE: International Centre of Insect Physiology and Ecology
ILRI: International Livestock Research Institute
KALRO: Kenya Agricultural and Livestock Research Organization
KENTTEC: Kenya Tsetse and Trypanosomiasis Eradication Council
OSF: Open Science Framework
PCR: Polymerase chain reaction
PCR-HRMA: Polymerase chain reaction-high-resolution melt analysis
PDF: Portable Document Format
PT: Precipitin test
qPCR: Quantitative polymerase chain reaction
SMRT: Single molecule real time
